# Crafting and Analyzing Multi-Structured Aramid Materials and Their Pyrolytic Transformations: A Comprehensive Exploration

**DOI:** 10.3390/polym15214315

**Published:** 2023-11-03

**Authors:** Miriam Trigo-López, Álvaro Miguel, José M. García, Aránzazu Mendía, Virginia Ruiz, Artur J. M. Valente, Saúl Vallejos

**Affiliations:** 1 Grupo de Polímeros, Departamento de Química, Facultad de Ciencias, Universidad de Burgos, Plaza de Misael Bañuelos s/n, 09001 Burgos, Spain; alvaro.miguel@ubu.es (Á.M.); jmiguel@ubu.es (J.M.G.); amendia@ubu.es (A.M.); 2 Facultad de Ciencias, Universidad Autónoma de Madrid, Calle Einstein 3, 28049 Madrid, Spain; 3 ProElectro, Departamento de Química, Facultad de Ciencias, Universidad de Burgos, Plaza de Misael Bañuelos s/n, 09001 Burgos, Spain; vrfernandez@ubu.es; 4 Department of Chemistry, Coimbra Chemistry Centre, University of Coimbra, 3004-535 Coimbra, Portugal; avalente@ci.uc.pt

**Keywords:** pyrolysis, aromatic polyamides, aramids, microporous-materials, Nomex, Kevlar, *meta*-aramid, *para*-aramid

## Abstract

Gradient porous materials, particularly carbon-based materials, hold immense potential in the fields of batteries, energy storage, electrocatalysis, and sensing, among others, by synergistically combining the attributes associated with each pore size within a unified structural framework. In this study, we developed a gradient porous aramid (GP-Aramid) by incorporating cellulose acetate as a porosity promoter in the polymer casting solution in different proportions. These GP-Aramids were subsequently transformed into their pyrolyzed counterparts (GP-Pyramids), retaining their original structures while displaying diverse cellular or dense microstructures inherited from the parent aramid, as confirmed via scanning electron microscopy. X-ray diffraction spectra provided evidence of the conversion of aramids into carbonaceous materials. The materials showed structural defects observed through the intensity ratio of the G and D bands (ID/IG = 1.05) in the Raman spectra, while X-ray photoelectron spectra (XPS) revealed that the carbonization process yielded pyrolyzed carbon materials unusually rich in nitrogen (6%), oxygen (20%), and carbon (72%), which is especially relevant for catalysis applications. The pyrolyzed materials showed bulk resistivities from 5.3 ± 0.3 to 34.2 ± 0.6 depending on the meta- or para-orientation of the aramid and the porous structure. This work contributes to understanding these gradient porous aromatic polyamides’ broader significance and potential applications in various fields.

## 1. Introduction

Functionally graded materials (FGMs) are materials that exhibit a gradual and continuous variation of their properties throughout their structure, enabling them to adapt and optimize for specific functions in different regions [1,2]. In this study, our primary objective was to synthesize carbon-like materials with a gradient porosity (a continuous material, not distinct layers) containing a high proportion of nitrogen and oxygen, employing a straightforward preparation method using aramids as the main materials and cellulose acetate (CA) as a porosity promoter, and employing a straightforward preparation method.

Unlike conventional materials, which have uniform properties throughout their volume, functionally graded materials are designed in such a way that properties intentionally vary, whether it be in terms of chemical composition, microstructure, density, mechanical strength, thermal conductivity, or other relevant properties. These gradual variations allow functionally graded materials to combine desirable characteristics from different materials into a single structure. They find applications in wear-resistant coatings, structural components in the aerospace industry, thermal insulation systems, electronics, and biomaterials [3,4,5,6].

The production methods for this type of materials are diverse, primarily depending on the chemical nature of the material. They can be prepared through deposition-based methods (vapor deposition, electrodeposition, thermal spray), solid-state methods (powder metallurgy methods, additive manufacturing methods, hybrid methods with additive manufacturing), and liquid-state methods (centrifugal force methods, slip casting methods, tape casting methods, infiltration methods, the Langmuir–Blodgett method) [6].

When the gradual and continuous variation refers to porosity, we refer to gradient porous materials [5,7], a field within materials science that has developed over the past 30 years [6], with titanium- and steel-based materials playing a prominent role [8,9].

However, moving away from metal-based gradient porous materials, some studies have focused on different polymers [10,11] and carbon-based materials due to their low cost and high availability [12,13]. In fact, certain authors have even prepared multilevel gradient structural carbon using naturally preprocessed biomass [14], or Lyocell [15], as precursors. These materials hold great promise in the field of batteries and energy storage, and studies have already been published where they are incorporated into methanol fuel cells [16]. Due to their physicochemical properties, such as high specific surface area, high thermal conductivity at room temperature, and a high Young’s modulus, carbon materials are also relevant in other fields such as electronic devices, sensors, and energy storage [17,18,19,20,21,22].

Graphene-like carbon materials can be synthesized through the pyrolysis of biomass [20], metal–organic frameworks (MOFs) [23,24,25], and polymers derived from natural sources [15,26,27]. Additionally, synthetic polymers containing heteroatoms, such as polypyrrole [28], polybenzoxazines [29], aromatic polyamides [30,31], or pyrimidine polymers [32], can also be utilized. The presence of heteroatoms doping the carbon material is common and, in some applications such as seawater desalination devices, flame retardant materials, electrochemical devices, environmental treatments, and pollutant removal, it is even essential [33,34].

Aromatic polyamide, specifically aramid, fibers such as poly(*m*-phenylene isophthalamide) and poly(*p*-phenylene terephthalamide), known by their commercial names Nomex^®^ and Kevlar^®^, respectively, have been utilized for the synthesis of N- and O-doped carbon materials through pyrolysis [30,35,36,37,38]. However, to the best of our knowledge, pyrolysis of these materials has only been conducted using fibers and textiles, resulting in the formation of nanometer-sized porous carbon materials after fiber pretreatment with H_3_PO_4_ or KOH, or through post-treatments involving activation with CO_2_, water vapor, or O_2_ [35,39,40].

This study elucidates a method for creating two innovative materials characterized by continuous gradient porosity. The initial material is grounded in aromatic polyamides (aramids), and the second material is produced through the pyrolysis of the former, yielding a chemically structured sp^2^ carbon material extensively doped with notable amounts of nitrogen (N) and oxygen (O), something that is rather unusual when compared to the pyrolysis of other polymer types.

**Figure 1:**
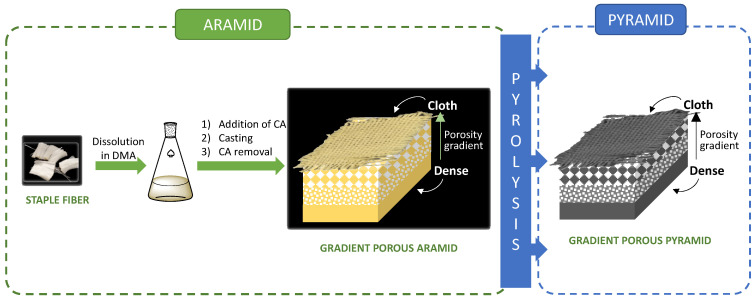
Table of contents.

We have pioneered the production of controlled porosity aromatic polyamides as conventional materials (not gradient porous materials), ranging from nanoporous to microporous structures, and using standard solution casting procedures. Furthermore, we have developed a scalable and cost-effective preparation procedure utilizing porosity promoter polymers [41,42,43]. Currently, we are introducing the production of gradient porous aramids (GP-Aramids) and gradient porous pyrolyzed aramids (GP-Pyramids), opening the door to their future applications. These materials have been prepared using both *m*-aramids and *p*-aramids in various formats and shapes, including continuous and discontinuous fabrics, as well as dense or porous films, as illustrated schematically in Figure 1.

GP-Aramids and GP-Pyramids can be prepared on a large scale with controlled porosities [44] without the loss of the structural integrity, and they can be key materials for energy, batteries, catalysis, etc., since these large porosities can provide large specific surface areas and porous pass-through channels to facilitate mass transfer. The materials have been characterized via Fourier transform infrared spectroscopy (FT-IR), RAMAN, scanning electron microscopy (SEM), X-ray photoelectron spectra (XPS), powder X-ray diffraction (PXRD), and electrical resistance.

## 2. Experimental

### 2.1. Materials

All materials and solvents were commercially available and used as received unless otherwise indicated. The following materials and solvents were used: acetone (99%, VWR), *N*,*N*-dimethylacetamide (DMAc) (99%, Sigma-Aldrich, Saint Louis, MO, USA), LiCl (≥99%, Sigma-Aldrich), cellulose acetate (CA) (40% wt. acetylated, 30 KDa, Sigma-Aldrich), poly(*p*-phenylene terephthalamide) (*p*-aramid; continuous fiber, average fiber diameter = 11.2 μm; textile, average yarn diameter = 13.2 μm; both calculated using SEM), poly(*m*-phenylene isophthalamide) (*m*-aramid; staple fiber; average fiber length and diameter of 6.4 mm and 13.2 μm, respectively, calculated using SEM).

Handling solvents like acetone and dimethylacetamide necessitates stringent safety measures. Ensure proper ventilation, use chemical-resistant gloves and safety goggles, and keep these solvents away from ignition sources. When using furnaces at temperatures as high as 800 °C, adhere to high-temperature equipment handling protocols, wear heat-resistant gloves and eye protection, and have suitable fire extinguishers on hand in case of a fire.

### 2.2. Instrumentation and Methods

The process of pyrolysis was conducted using a thermobalance instrument (Q50 TGA analyzer, TA Instruments, New Castle, DE, USA).

The infrared spectra (FTIR) of the aramids were captured using an infrared spectrometer (FT/IR-4200, Jasco, Tokyo, Japan) equipped with an ATR-PRO410-S single reflection accessory. To obtain the FTIR transmittance spectra of the aramids, the samples were first pulverized and then incorporated into KBr pellets.

Scanning electron microscopy (SEM) investigations were conducted using an electron microscope (FEI Quanta 600, FELMI-ZFE, Graz, Austria). The aromatic polyamides underwent a drying process in ambient air, followed by fracturing and gold sputtering in a vacuum to ensure electrical conductivity. However, it is worth noting that no gold sputtering was applied to the pyramids, as they are inherently conductive materials.

Raman spectra were obtained using a confocal AFM-Raman system, specifically the Alpha300R—Alpha300A AFM by WITec, located in Ulm, Germany. The measurements were conducted with laser radiation at 532 nm and a power output of 1 mW, with a magnification set at 100×.

Sample electrical resistance (*R*) was quantified using a Jandel 4-point probe (HL Jandel, UK) linked to a 2401 SourceMeter^®^ unit (SMU) instrument from Keithley, OH, USA. The surface bulk resistivity (ρb) of the samples was subsequently determined using the following formula:

ρ_b_ = 2π*sR* (1)

In this equation, ‘*s*’ represents the electrode spacing, which is fixed at 0.125 cm [45]. As references, the electrical resistance of both graphite and steel samples was also measured and taken into account.

Powder X-ray diffraction (PXRD) patterns were acquired utilizing a diffractometer (D8 Discover Davinci design, Bruker Corporation, Billerica, MA, USA). The instrument operated at 40 kV and employed Cu(Kα) as the radiation source. The scan step size was set at 0.02°, and each scan step took 2 s to complete.

X-ray photoelectron spectra (XPS) were captured using a Fisons MT500 spectrometer equipped with a hemispherical electron analyzer known as CLAM2. A non-monochromatic Mg Kα X-ray source operating at 300 W was employed for excitation. The samples were securely affixed to small flat discs supported on an XYZ manipulator, strategically positioned within the analysis chamber. The ion-pumped analysis chamber was rigorously maintained at a residual pressure below 10^−9^ torr throughout the data acquisition process. Spectra were recorded with a pass energy of 20 eV, a setting typically used for high-resolution conditions. Subsequently, CasaXPS 2.3.25 software was employed for spectrum analysis. To estimate intensities, the area beneath each peak was calculated after removing the S-shaped background, and the experimental curve was fitted using a combination of Lorentzian and Gaussian lines with adjustable proportions. It is worth noting that despite the presence of specimen charging, accurate binding energies (BEs) were determined with reference to the adventitious C1s peak at 285.0 eV. The allowable variation in binding energy was limited to ±0.2 eV relative to the specified peak center value. Atomic ratios were computed from the intensity ratios of the peaks using established atomic sensitivity factors, as reported [46].

### 2.3. Preparation of GP-Aramids

GP-Aramids were prepared using a standard solution casting procedure. The region with zero porosity (dense zone) of the material was prepared by carrying out a casting process with a solution containing 0.49 g of commercial *m*-aramid, 0.14 g of LiCl (as a solubility promoter), and 7 mL of DMAc (the solution is prepared at 80 °C). A total of 5 mL of this solution was poured onto a circular silicone mold, and the system was placed in an air-circulating oven at 80 °C for 4 h. Then, the addition and evaporation process was repeated two more times.

After the initial region, the porosity gradient was controlled by incorporating cellulose acetate as a porosity promoter. In previous studies, we have examined various porosity promoters, such as ionic liquids [41,43,44], boron nitride [47], and different polymers [42], including polyvinyl alcohol (PVA), poly(2-ethyl-2-oxazoline) (PEOx), and cellulose polyacetate (CA). For this work, we have chosen CA, as it allows for excellent control of porosity and is also cost-effective. Using the same experimental conditions, the low-porosity region was prepared using a solution composed of 0.245 g of cellulose acetate, 0.49 g of commercial *m*-aramid, 0.14 g of LiCl, and 7 mL of DMAc. The high-porosity region was prepared using a solution consisting of 0.49 g of cellulose acetate, 0.49 g of commercial *m*-aramid, 0.14 g of LiCl, and 7 mL of DMAc. A total of 3 mL instead 5 mL of this solution was poured onto the circular silicone mold. Finally, a layer of woven *p*-aramid fabric was added to include a region with extremely high porosity. The fabric was soaked with DMAc to ensure cohesion with the layer underneath.

After that, the materials were washed with water and, via Soxhlet extraction, with acetone for 48 h to remove the porosity promoter polymer (CA) and generate the GP-Aramids.

In order to accurately characterize the different porosity regions (continuous material, different regions), each of them was also prepared as an individual material (separate layers) following the same experimental procedure.

### 2.4. Pyrolysis of Aramids

For the preparation of GP-Pyramids, GP-aramids were pyrolyzed using a thermobalance, and applying a nitrogen current of 40 mL/min. The samples were heated at 2 °C/min up to 800 °C, and finally, an isothermal reaction was carried out for 5 h. The samples were cooled using the same nitrogen gas flow until the temperature reached below 150 °C (around 2 h).

## 3. Results and Discussion

**Figure 2:**
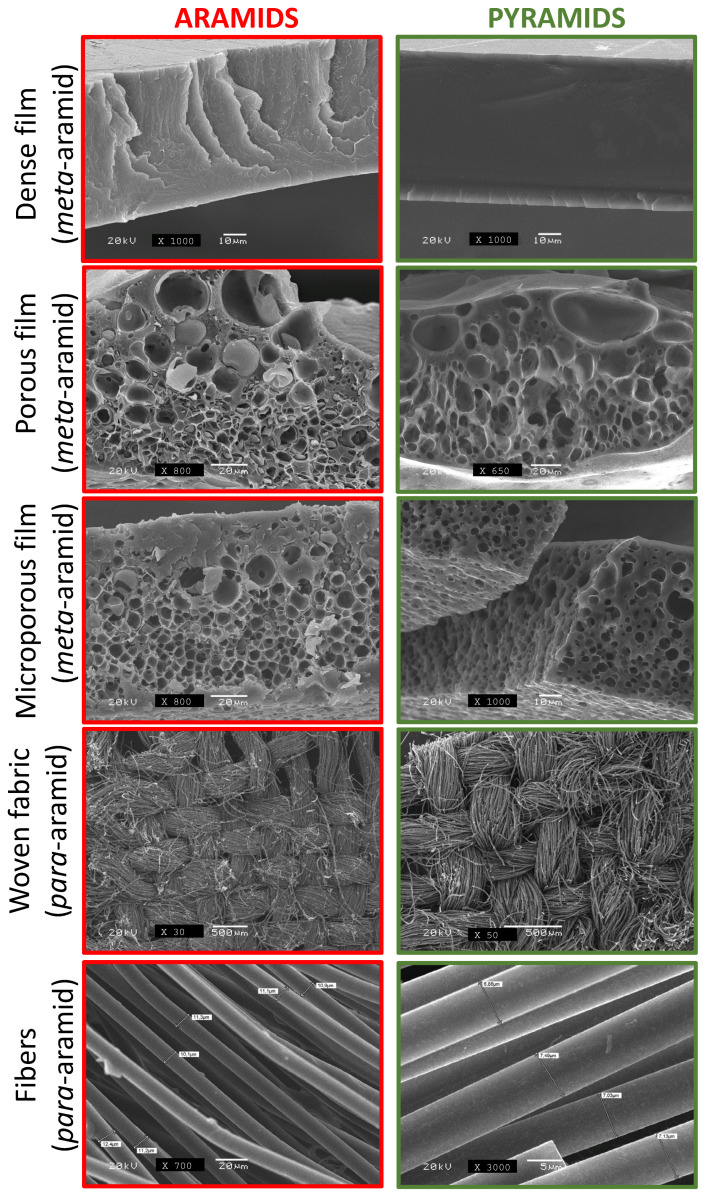
SEM images of aramids and pyramids in different formats (dense film, porous film, microporous film, woven fabric, and fibers).

In our previous research [41,42,43,44], we successfully fabricated porous *m*-aramids with various cellular morphologies by incorporating a porosity promoter polymer in the casting process. The objective of our current study was to produce porous aramids using high percentages of cellulose acetate (CA) (50% and 100% relative to the aramid) and investigate whether their pyrolysis yields conductive materials while preserving the cellular structure. Additionally, we also pyrolyzed dense *m*-aramid films and *p*-aramid fibers and textiles in the same conditions for characterization purposes. Our findings demonstrate that we can indeed generate conductive materials while maintaining their original shape and cellular structure, as observed in MOFs [48]. Figure 2 presents scanning electron microscopy (SEM) micrographs of the various individual materials, including *m*-aramids and *p*-aramids (dense, porous, microporous, woven fabric, and fibers), before and after the pyrolysis procedure, illustrating the structural integrity post-process. The complete investigation involved the use of two varieties of aromatic polyamide featuring amide bonds oriented in both the *meta*- and *para*-positions.

**Figure 3:**
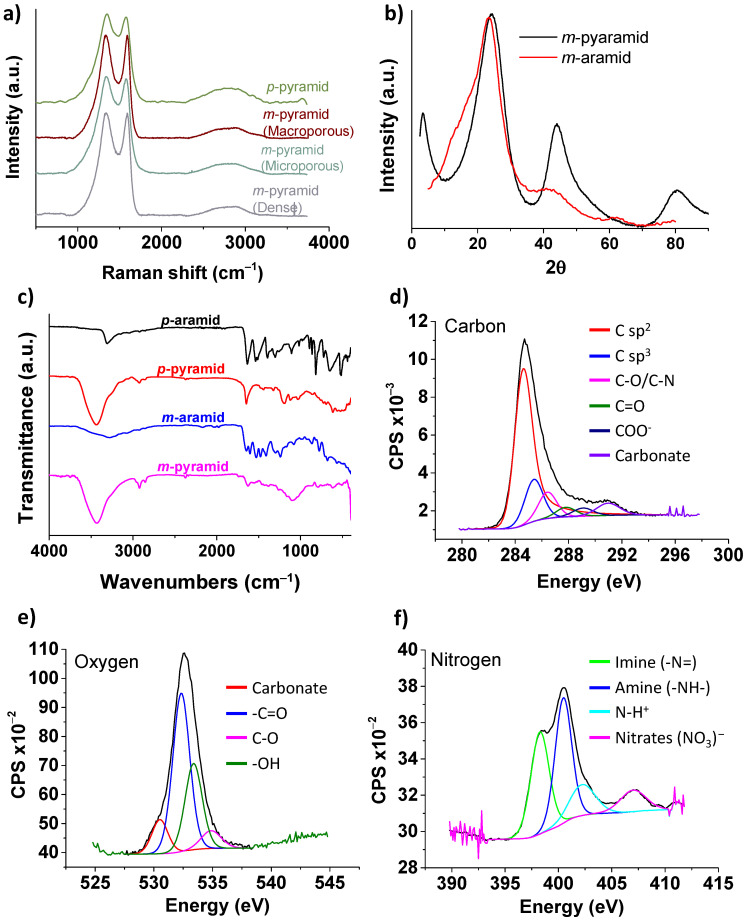
(**a**) Raman spectra of *meta*- and *para*-pyramids. (**b**) XRD spectra of *m*-aramid and *m*-pyramid. (**c**) FTIR of *m*- and *p*-aramids and *m*- and *p*-pyramids. (**d**–**f**) XPS high-resolution spectra corresponding to carbon, oxygen, and nitrogen, respectively, in dense *m*-pyramid.

Raman spectroscopy, as depicted in Figure 3a, confirmed the existence of defects in the carbonaceous structure. The G band, with an intensity at 1590 cm^−1^, represents the characteristic stretching vibrational mode of sp^2^-hybridized carbon in a graphitic 2D hexagonal lattice. It serves as the primary mode in graphene and graphite. The D band, observed at 1350 cm^−1^, is referred to as the defect/disordered band. It is attributed to a breathing vibrational mode originating from aromatic sp^2^ carbon located adjacent to layer edges or defects. Consequently, the D band is not discernible in defect-free pristine graphene. The evaluation of the intensity ratio between these vibrational modes is commonly employed to quantify the defect content in graphene-like materials. In our case, the ID/IG ratio is 1.05, indicating the presence of defects in the material. According to the literature, this value aligns with the “mainly sp^2^ amorphous carbon” regime typically associated with graphene oxide [49,50].

The high density of defects observed in the pyrolyzed materials can be attributed to the carbonization process, which follows a reaction mechanism similar to that of polyacrylonitrile. Around 500–600 °C, aryl nitriles are formed, followed by aromatization and condensation reactions at higher temperatures, with a notable retention of heteroatoms (oxygen and nitrogen) [51,52]. Hence, one of the primary objectives of this work was to demonstrate our ability to maintain these high levels of oxygen and nitrogen in our materials. XPS analysis (Figure 3d–f) revealed that the atomic content of nitrogen, oxygen, and carbon in the pyrolyzed *m*-oriented aramid was approximately 6%, 20%, and 72%, respectively. The remaining 2% is attributed to elements such as Si and Cl, which we associate with a process that occurred in the molds used for material preparation. These molds are treated with a 15% solution of dichlorodimethylsilane in toluene to enhance their anti-adhesive properties, thus facilitating the demolding process of the prepared materials.

The high amount of nitrogen and oxygen present is one of the key points of our study, as it encompasses not only the attainment of a carbon structure with a porosity gradient but also a high percentage of nitrogen and carbon. This, in turn, broadens the range of potential applications for these materials. Oxygen is likely associated with the aromatic network in pyridones, quinones, or similar forms, as well as hydroxyl and carboxylic functional groups. Nitrogen exists in chemically stable aromatic forms, with one third as imine-type nitrogen, one third as amine-type nitrogen, and the remaining one third as nitrates. Most of the carbon content is in the form of sp^2^ carbon, but sp^3^ hybridization is also present. These functional groups in the pyrolyzed materials are consistent with the observations in the FTIR analysis (Figure 3c), which show stretching bands at 3435 cm^−1^ for -OH groups, 2923 cm^−1^ for -CH_2_, and the absence of the C=O stretching band from the amide group. The PXRD spectra of *m*-aramid and *m*-pyramid also indicate the transformation of the aramid into a carbonaceous material (Figure 3b), with two broad diffraction peaks corresponding to the (002) and (100) diffraction peaks at 2θ = 22° and 45°, respectively [37,53].

The surface electrical conductivity of the samples was evaluated. It has been confirmed that aramids are essentially non-conductive. However, the *p*-pyramids exhibited a bulk resistivity of 5.3 ± 0.3 Ω·cm, which is comparable to the measured resistivity of graphite (3.6 ± 0.2 Ω·cm) or steel (0.71 ± 0.01 Ω·cm). Interestingly, an increase in bulk resistivity was observed in the case of *m*-pyramids as the porosity increased (7.5 ± 0.4, 9 ± 2, and 34.2 ± 0.6 Ω·cm for dense, microporous, and porous *m*-pyramids, respectively). This increase in resistivity could be attributed to the loss of interchain electron mobility caused by the increased porosity [54]. These findings suggest that the electrical conductivity of the materials is influenced by their chemical composition and the presence of pores. Although the observed differences are not substantial, these results provide valuable insights into the fundamental properties of the materials and their potential suitability for various applications, including coatings (to be employed in photocatalysis, magnetic drug delivery systems, rechargeable Li-ion batteries, etc.) [55], solar cells (carbon-based cells could potentially offer a more cost-effective alternative to silicon-based solar cells, with significantly lower costs and superior chemical and environmental stability when compared to silicon) [56], and adsorbents (our carbonaceous materials have high specific surface areas and strong π–π interactions) [57].

**Figure 4:**
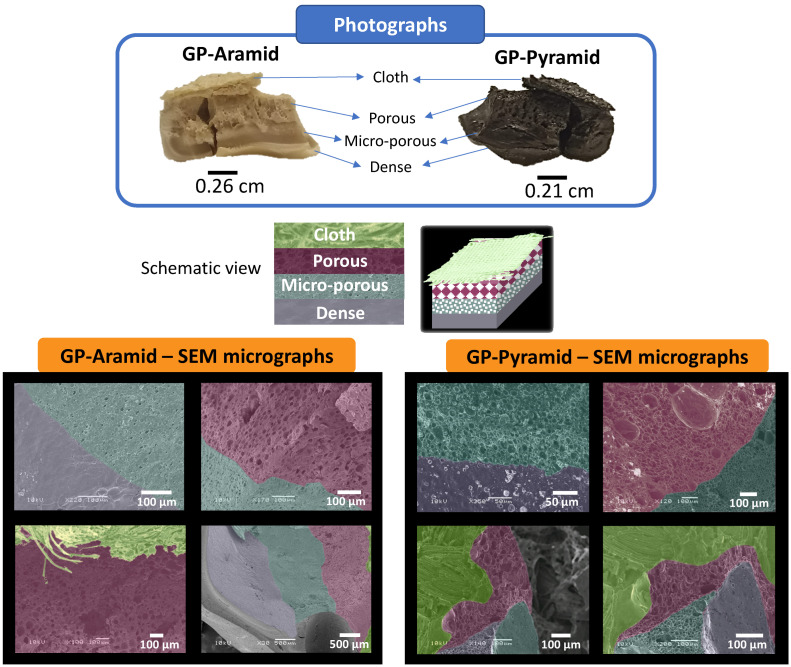
SEM images and photographs of the gradient porosity materials GP-Aramid and GP-Pyramid.

Finally, we achieved the aim of our study by successfully fabricating gradient porosity materials, namely GP-Aramids and GP-Pyramids. The materials were composed of an initial dense layer, followed by a microporous layer, a porous layer, and, to include an extremely highly porous layer, a fabric layer. Figure 4 showcases the acquired structures through scanning electron microscopy (SEM) images and photographs. The gradient porosity could be tailored according to specific requirements by adjusting the quantity of the porosity promoter, cellulose acetate (CA). This figure effectively underscores the adaptability and precision of the employed fabrication technique in creating these remarkable structures. We have encountered substantial difficulties in the process of measuring the mechanical properties of our pyrolyzed materials. The universal testing machines tend to damage the material when it is gripped by their jaws. Consequently, we intend to conduct further research on this matter in forthcoming studies.

## 4. Conclusions

In conclusion, our study represents a significant step forward in the realm of materials science. By exploring the potential of gradient porosity aramids and their seamless transformation into carbonaceous materials with retained porosity during pyrolysis, we have not only expanded the toolbox for material design but also unlocked a spectrum of promising applications. Our key achievements and implications can be summarized as follows. (1) Structural stability and customizability: We have demonstrated the remarkable structural stability of gradient porosity aramids throughout the pyrolysis process, which offer a unique advantage in creating materials with customizable porosity levels. This versatility empowers researchers and designers to tailor materials to specific applications. (2) Electrocatalytic potential: The substantial nitrogen content, notably in the form of pyridine-N, highlights the electrocatalytic potential of these materials. They hold promise for applications such as the oxygen reduction reaction in fuel cells and metal–air batteries, marking a substantial advancement in sustainable energy technologies. (3) Sensitivity to gases: These materials exhibit an enhanced affinity for gases like CO, NO, and NO_2_, making them valuable candidates for sensor applications. Their substantial alterations in electronic properties in response to these gases open up avenues for improved gas sensing technologies. (4) Catalytic exploration: By introducing metals such as iridium or palladium through in situ reduction during pyrolysis, we could unveil their potential in catalytic reactions, particularly hydrogenation. This paves the way for innovations in catalysis and green chemistry. (5) Circular economy contribution: Repurposing waste materials from police and firefighter clothing (mainly composed of *meta*-aramid) as a source of recycled raw material offers an eco-friendly solution to mitigate the high costs associated with aramids. This aligns with the principles of the circular economy, contributing to sustainability and resource efficiency. In light of these accomplishments, our study not only advances the field of materials science but also holds great promise for addressing pressing challenges in sustainable technology, catalysis, and circular economy initiatives. It underscores the importance of considering the potential of unconventional materials and processes in our quest for innovative solutions.

## Data Availability

The raw data required to reproduce these findings are available to download from https://riubu.ubu.es/handle/10259/5684 (accessed on 11 February 2023) (dataset of the work “Crafting and Analyzing Multi-Structured Aramid Materials and Their Pyrolytic Transformations: A Comprehensive Exploration”).
